# Transforming hematological research documentation with large language models: an approach to scientific writing and data analysis

**DOI:** 10.1007/s44313-025-00062-w

**Published:** 2025-03-06

**Authors:** John Jeongseok Yang, Sang-Hyun Hwang

**Affiliations:** 1 Department of Laboratory Medicine and Genetics, Samsung Medical Center, Sungkyunkwan University School of Medicine, Seoul, 06351 Republic of Korea; 2 Department of Laboratory Medicine, Asan Medical Center, University of Ulsan College of Medicine, Seoul, 05505 Republic of Korea; 3 Asan Medical Center, Asan Institute for Life Sciences, University of Ulsan College of Medicine, Seoul, 05505 Republic of Korea

**Keywords:** Large language models, Hematology research, Scientific writing, Prompt engineering, Medical research, Artificial intelligence

## Abstract

Large Language Models (LLMs), such as ChatGPT (OpenAI, CA, US), have revolutionized scientific writing and research processes across academic disciplines, providing comprehensive support throughout the entire research lifecycle. Generative artificial intelligence (GAI) tools enhance every aspect of scientific writing, from hypothesis generation and methodology design to data analysis and manuscript preparation. This review examines the applications of LLMs in hematological research, with particular emphasis on advanced techniques, including prompt engineering and retrieval augmented generation (RAG) frameworks. Prompt engineering methods, including zero-shot and few-shot learning along with a chain-of-thought approach, enable researchers to generate more precise context-specific content, especially in scientific writing. Integrating RAG frameworks with the current medical literature and clinical guidelines significantly reduces the risk of misinformation while ensuring alignment with contemporary medical standards. Even though these GAI tools offer remarkable potential for streamlining research writing and enhancing documentation quality, the study also addresses the critical importance of maintaining scientific integrity, ethical considerations, and privacy concerns in hematological research.

**Supplementary Information:**

The online version contains supplementary material available at 10.1007/s44313-025-00062-w.

## Introduction

Large Language Models (LLMs) are a subset of generative artificial intelligence (GAI); in this review, these terms are used interchangeably without distinction. These models can *learn* complex language patterns and grammatical relationships through extensive training processes on vast amounts of text data, allowing them to *understand* and generate text. Natural language processing has been revolutionized with the advent of LLMs, such as Bidirectional Encoder Representations from Transformers (BERT) [1] and the Generative Pre-trained Transformer-4 (GPT-4) model (OpenAI, CA, US) [2], building upon the groundbreaking development of transformer models in 2017 [3]. The development of advanced models like BERT and iterations of OpenAI's GPTs has significantly improved human-like text generation and contextual understanding, transforming content creation, grammar enhancement, citation management, and real-time feedback [1, 4].

The accessibility of GAI tools was significantly broadened by the release of ChatGPT in late 2022, profoundly impacting researchers and healthcare professionals [5]. LLMs have catalyzed numerous innovations in the medical domain, including medical documentation [6], clinical decision support [7], and enhanced patient communication [8]. Moreover, LLMs are being applied in hematology for various purposes, including medical education [9], diagnosis of leukemia [10]**,** hemoglobinopathies [11], improving clinical practice, assisting in predicting drug responses [12], and analyzing clinical data [13, 14].

However, the process of crafting a high-quality scientific manuscript is often time-intensive and requires meticulous attention to detail and adherence to journal-specific guidelines. Recently, several researchers have directly utilized LLMs in their research and writing processes [15]. The integration of LLMs into manuscript writing extends beyond grammar enhancement and multilingual translation [16]. Advanced GAI models capable of inference, including OpenAI's ChatGPT and Anthropic's Claude, have demonstrated the capability to generate new research ideas, synthesize large volumes of information, and assist with literature reviews. They can also suggest statistical tests and research directions based on existing data and contribute to the full scientific discovery and writing process, including hypothesis generation, experiment planning, manuscript drafting, and peer review [17–19]. These models significantly improve accuracy through grammar and style error reduction [20]. Their multilingual capabilities support non-English-speaking researchers, facilitate enhanced research collaboration and knowledge sharing, and exhibit features aligned with human judgments on grammatical construction [21].

Despite these advancements, achieving optimal LLM performance in scientific writing requires considerable strategic interaction. In such cases, prompt engineering is a prerequisite. Prompt engineering is an emerging discipline that focuses on the systematic design and optimization of contextually relevant prompts, which are crucial for enhancing the efficacy of LLMs by improving their performance and ensuring the generation of accurate domain-specific outputs across diverse scientific and medical applications. A well-structured prompt serves as a crucial tool in effectively directing LLMs to generate precise, relevant, and insightful content tailored to specific needs. Various prompting techniques, including few-shot prompting [22], chain-of-thought (CoT) prompting, and self-consistency strategies, have been developed to optimize the use of LLMs in scientific research and writing [23].

In addition to prompt engineering, Retrieval-Augmented Generation (RAG) enhances LLM performance by integrating the generative capabilities with real-time access to external knowledge sources [24]. RAG systems dynamically retrieve information from verified databases, scientific literature, and institutional repositories, allowing researchers to incorporate the most current and relevant data into their writing [25]. This approach mitigates the limitations of LLMs that rely solely on pre-trained knowledge and ensures factual accuracy, contextual relevance, and compliance with the latest scientific standards. By integrating RAG with LLM-driven scientific writing, researchers can effectively bridge knowledge gaps, validate their findings using authoritative sources, and improve the overall credibility and trustworthiness of their manuscripts. This study provides a brief overview of the current status of GAI in scientific writing, highlighting its benefits, limitations, and potential future applications in the research publication process.

### Prompt engineering and RAG in hematological context

**Fig. 1:**
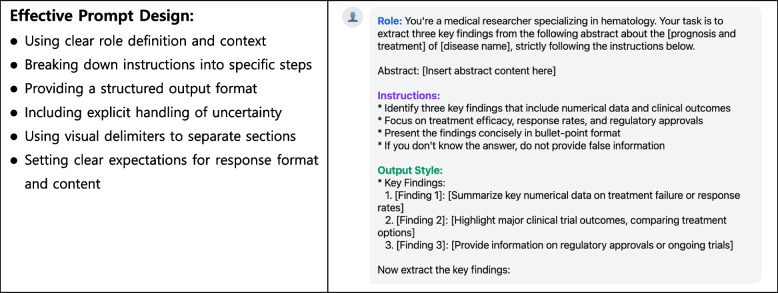
Representative example prompt: extracting key findings from abstracts. Example of a structured prompt template demonstrating how to systematically extract key findings from medical literature. The prompt includes three essential components: (1) Role definition establishing medical research context, (2) Clear instructions for data extraction with a specific focus on numerical outcomes and clinical data, and (3) structured output format guidelines. This template illustrates effective prompt engineering practices for literature review and research synthesis in medical research contexts

In LLMs, a prompt refers to a set of instructions or queries provided by users to guide the output of a model. This includes a wide range of text styles ranging from simple questions to detailed explanations or specific tasks [26]. The elements of a prompt include the instructions, context, input data, and output [27]. To achieve an effective prompt design, providing clear and specific instructions, using delimiters to separate distinct parts of the input, employing structured output formats, prompting the model to consider its prior responses, breaking down complex tasks into simple steps, and specifying the desired tone and style is essential (Fig. 1) [28, 29].

**Fig. 2:**
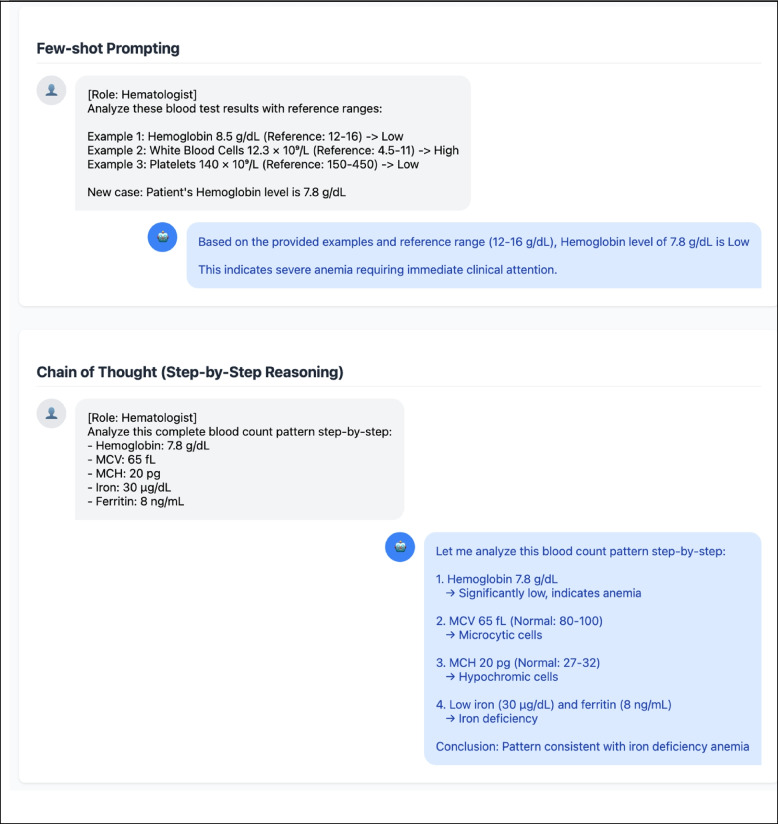
Demonstration of various prompting strategies with clinical examples. Visual representation of two key prompting strategies employed in medical analysis. **A** Few-shot prompting strategy demonstrates how providing relevant examples guides artificial intelligence in analyzing laboratory test results with specific reference ranges. **B** The chain-of-thought (Step-by-step reasoning) approach demonstrates a systematic analysis of clinical data patterns for diagnostic reasoning. This visualization is generated using Claude 3.5 Sonnet and implemented with HTML, CSS, and JavaScript. The complete source code is available via Online Resource 1 (Supplementary Table S1)

Prompt engineering represents a sophisticated approach for optimizing the output of LLMs through the strategic design and structuring of input prompts [30]. Prompt engineering encompasses a variety of techniques including few-shot prompting and COT. Few-shot prompting is a well-known and effective strategy that leverages the pre-trained knowledge of a model by providing two to three carefully selected examples to establish the desired context and output format [22]. This approach enables efficient task completion, making it particularly valuable for applications requiring consistency across large volumes of content (Fig. 2).

Furthermore, COT prompting offers a sophisticated approach by explicitly articulating logical reasoning processes in a step-by-step manner to generate customized outputs [23]. This methodology is particularly effective when dealing with complex concepts or high-difficulty tasks that require detailed explanations and nuanced understanding. By breaking down complex tasks into modular prompt blocks and incrementally building COT reasoning, researchers can achieve more precise and targeted results [31] (Fig. 2).. Upon providing a specific guideline, outputs can determine whether the given input meets the criteria for a certain type of anemia and recommend the necessary testing for confirmation [32].

Additional prompting strategies have been developed to meet the unique needs of different contexts. Zero-shot prompting eliminates the need for examples by providing clear and detailed instructions that enable the models to perform tasks without prior examples [33]. Role-based prompting assigns distinct personas or expertise levels to the model, thereby enhancing the appropriateness and specificity of the responses for specific domains. These diverse approaches can be strategically combined to develop hybrid prompting strategies that optimize the performance for specific use cases while maintaining efficiency and accuracy.

Quality assurance in prompt engineering is crucial for maintaining scientific rigor. Researchers should implement systematic validation processes, including the cross-referencing of primary sources, verification of statistical analyses, and adherence to field-specific standards. This process should be iterative, with continuous refinement of prompts based on output quality and accuracy. The documentation of the prompt development and validation steps ensured reproducibility and transparency in the research process.

The future of prompt engineering in hematological research lies in the development of increasingly sophisticated approaches that leverage advances in LLM technology while maintaining scientific integrity. As these models evolve, researchers must adapt their engineering strategies to leverage new capabilities while ensuring rigorous validation and quality control.

When an LLM hallucinates, it generates information that seems accurate but is actually false [34]. To reduce hallucinations and ensure a reliable LLM in production, practical prompting strategies include explicitly instructing the model not to provide false or unverifiable information; for instance, “If you don’t know the answer to a question, please don’t provide false information.” (Table 1).

**Table 1:** Prompt engineering strategies to reduce LLM hallucinations

Strategy	Description	Example Prompt
Explicit Instruction	Instruct the LLM directly to avoid uncertain information	“If you don’t know the answer, do not provide false information.”
Emphasizing Honesty	Encourage the LLM to acknowledge its knowledge limits	“If unsure, say ‘I don't know’.”
Requiring Verifiability	Emphasize the need for verifiable information	“Provide only information that can be verified by reliable sources”
Allowing Uncertainty Expression	Allow the model to express uncertainty	“State if you are unsure about the answer.”
Providing Context	Offer relevant background information for improved accuracy	“Consider this background before responding.”

Directly employing LLMs in academic writing can lead to issues such as hallucinations, in which the generated content is not established on factual data and fails to accurately reflect state-of-the-art research. Moreover, ChatGPT 4.0, demonstrated an accuracy range of approximately 43% to 87% in the general medical domain, depending on the complexity of the task and evaluation criteria [2, 35, 36]. To address these issues, numerous studies have been performed on the principle of RAG [37], which enhances LLM-based literature review generation by grounding in factual content retrieved from external sources, such as databases, guidelines, or high-quality journals. This approach enables the accurate retrieval of medical domain knowledge without requiring additional fine-tuning of the model.

A key feature of LLMs is their ability to process documents in various formats. Researchers can upload documents of interest directly, including PDF, word-processor documents, text files, and audio files. By providing models with queries for specific details, such as methodologies and experimental protocols, researchers can receive concise and clear explanations. This approach facilitates quick comprehension of the content, saving valuable time during the research process.

### LLM-driven data analysis in hematology: a prompt-based approach

The integration of LLMs with no-code platforms simplifies the academic research process, particularly for researchers with limited code-writing expertise. The popularity of LLMs, including GPT-4, Gemini, and Claude, has surged, establishing them as powerful and indispensable tools for writing Python scripts and handling various coding tasks, supporting computation-based research, and allowing researchers to work efficiently without profound programming knowledge. Their ability to explain workflows, identify errors, and interpret complex concepts, particularly in machine-learning applications, makes them valuable tools for researchers who may not be well-versed in code writing or command-line interfaces.

The advent of LLMs has proven their usefulness in accelerating data analysis research. LLMs can detect emphasized important findings from input data or queries, relieving researchers from redundant and laborious data analyses [38]. Additionally, LLMs can instantaneously produce publication-ready outputs in table or figure format. Furthermore, LLMs, such as ChatGPT and Claude, can assist in developing analysis code, debugging, and visualization, and are being integrated into productivity software(s) used for manuscript writing and presentation.

Data analysis in hematological research presents unique challenges that can be addressed through carefully crafted prompts and the corresponding code implementation. The complexity of hematological data, which often incorporates multiple parameters and time-series measurements, requires a nuanced approach that combines prompt engineering with computational analysis. A publicly available acute myeloid leukemia (AML) gene expression dataset comprising 405 patients was used to illustrate the data analysis workflows powered by LLMs for survival prediction [39]. The dataset was accessed through a publicly available repository in FigShare (ID: 13,585,235; https://figshare.com/s/7c683384c6e2add08262).

**Fig. 3:**
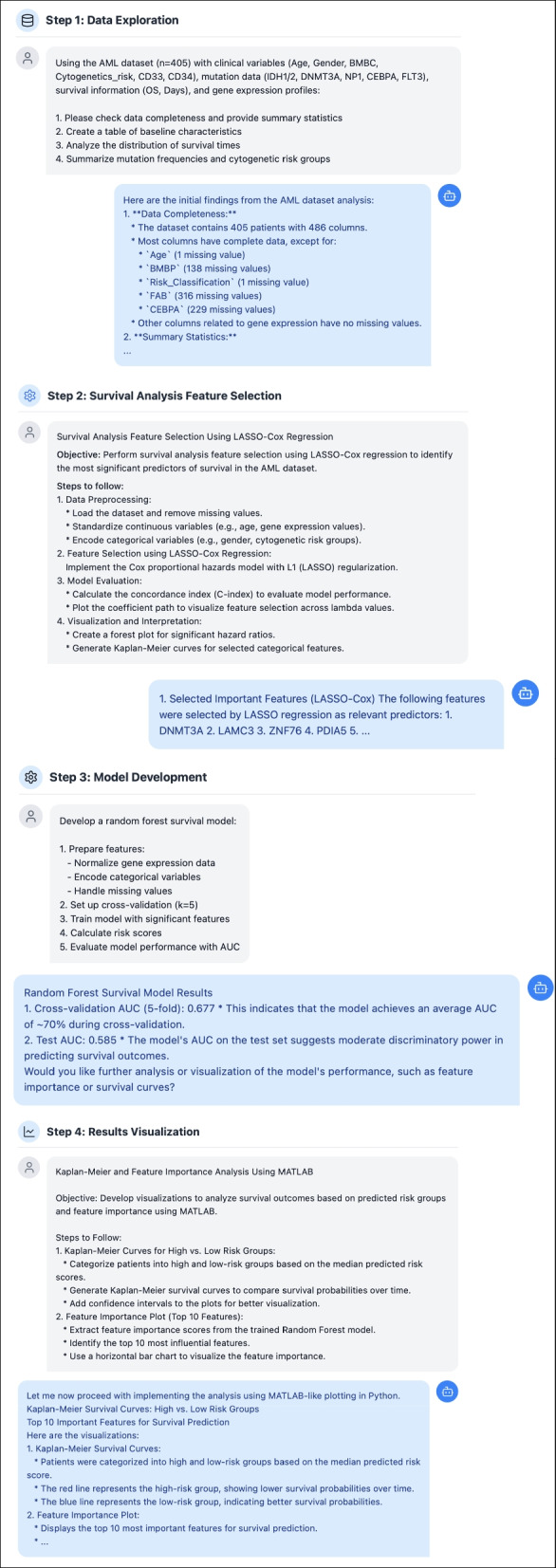
Prompt engineering framework for acute myeloid leukemia survival analysis. A systematic approach to prompt engineering is demonstrated through a conversational interface. The framework consists of four key steps: (**A**) Data Exploration—examining clinical variables, mutation data, and survival information; (**B**) Feature Selection—identifying significant prognostic features through univariate and multivariate analyses; (**C**) Model Development—implementing random forest survival prediction model; and (**D**) Results Visualization—creating survival curves and performance metrics. Each step illustrates the structured prompts (researcher) and expected responses (assistant). The visualization demonstrates the practical application of prompt engineering in survival analysis research. The provided examples serve demonstration purposes only. This visualization is generated using Claude 3.5 Sonnet and implemented with HTML, CSS, and JavaScript. The complete conversation is provided in Online Resource 2 (Supplementary Table S2) and the complete source code in Online Resource 3 (Supplementary Table S3). The results of the LASSO-Cox regression analysis may vary due to the absence of a fixed random seed, which affects feature selection consistency, and differences in software library versions across computational environments, leading to variations in algorithm implementation and numerical precision. Queries and prompts demonstrated here are for demonstration purposes, and users bear the final responsibility for verifying the generated results

Figure 3 illustrates a systematic approach for analyzing patients with AML data using LLMs to demonstrate prompt engineering strategies. The framework consists of four key steps: data exploration, feature selection, model development, and result visualization. During the data exploration phase, a dataset of 405 patients with AML was explored, including clinical variables, mutation data, and gene expression profiles. The feature selection utilized LASSO-Cox (Least Absolute Shrinkage and Selection Operator-Cox Proportional Hazards Model) regression to identify significant prognostic factors, while model development employed random forest techniques achieving a cross-validation area under the curve (AUC) of 0.6777 and test AUC of 0.585 (Fig. 3). Considering that the prompts and queries presented throughout the article are for demonstration purposes, all generated outputs and results are subject to thorough verification by the user.

Even though the examples provided are for demonstration purposes only, LLMs offer significant advantages in complex statistical analyses. However, code execution may not be error-free on the first attempt, and repeated queries, despite using the same prompts, may not be reproduced in identical styling. Additionally, a risk of missing statistical parameters, variables, or errors due to hallucinations exists. The recommended approach is to have LLMs generate the code but execute and validate the results in professional integrated development environments (IDEs) such as RStudio (Posit PBC, Massachusetts, USA), Visual Studio Code (Microsoft Corporation, Washington, USA), or Google Colab (Google LLC, California, USA). The latest versions of IDEs with artificial intelligence (AI) functions based on LLMs integrated natively into programs demonstrate how these models have become essential parts of the process.

### Requesting LLM-assisted framework for analysis

A structured approach, such as requesting step-by-step guidance on frameworks, pipelines, and systematic processes, is highly effective for tasks beyond data analysis, including scientific writing, personal work management, and project execution. By following this approach, one can ensure a significantly organized and efficient workflow, leading to improved productivity and better outcomes. This method offers a roadmap for tackling complex scans, ensuring consistent progress, and improving reproducibility across different domains.

**Fig. 4:**
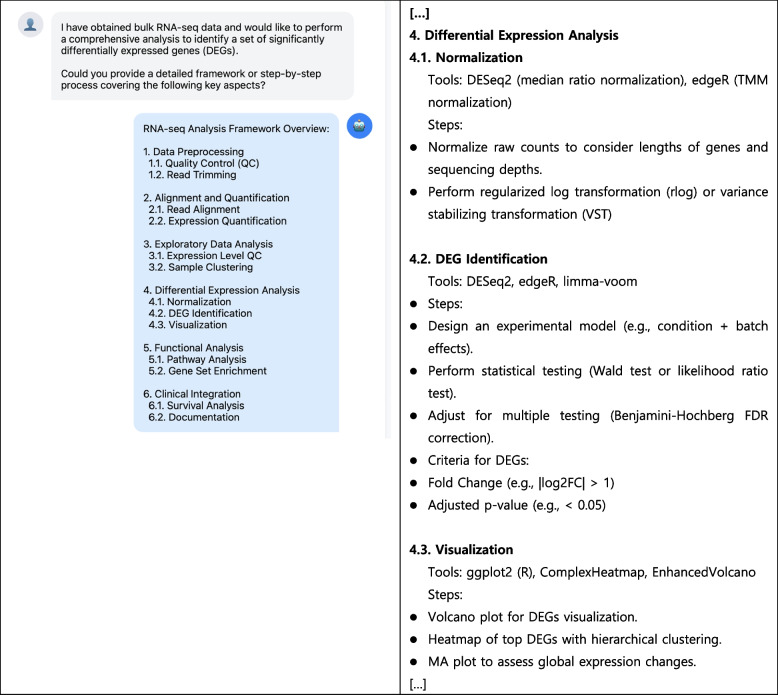
Large Language Models (LLM)-guided framework for ribonucleic acid sequence (RNA-seq) analysis in hematological research. Demonstration of prompting strategy for RNA-seq differential expression analysis framework development. Left; The conversation demonstrates a researcher's request for an RNA-seq analysis framework and LLM's structured response outlining key analytical stages from preprocessing to clinical integration. Right; Detailed description of RNA-seq analysis step 4 (complete dialogue available in Supplementary Table S4). The framework demonstrates how LLMs can effectively guide researchers through complex bioinformatics workflows while maintaining a focus on clinically relevant outcomes in hematological research. This visualization is generated using Claude 3.5 Sonnet with HTML/CSS/JavaScript

Research on hematological malignancies requires complex analytical pipelines to process diverse data types, including genetic, molecular, and clinical information. LLMs can streamline the development of analytical frameworks, particularly for integrating multiple data modalities. We demonstrated this capability by requesting a framework for differential gene expression analysis of AML ribonucleic acid sequencing data (Fig. 4).

### AI tools and resources for scientific writing in hematological research

LLMs are capable of translating languages and summarizing text information, allowing users to handle an expanded number of literature reviews efficiently [40]. A manuscript discussion typically starts by summarizing the key findings and contextualizing them within the existing literature. Although LLMs, such as ChatGPT and Claude Excel, summarize research findings, their ability to effectively review and cite relevant literature remains limited. ChatGPT or Claude may generate inaccurate citations or references to nonexistent publications owing to training limitations or hallucinations. Although the web browsing functions of ChatGPT and Perplexity (Perplexity AI Inc., San Francisco, CA, USA) offer certain improvements in literature search capabilities, they are restricted to web-accessible content limited by their model training period, potentially overlooking crucial publications. Therefore, a robust approach involves researchers conducting primary literature searches using established databases such as PubMed, Google Scholar, and Web of Science, followed by the use of LLMs to assist in analyzing and summarizing the selected publications.

**Fig. 5:**
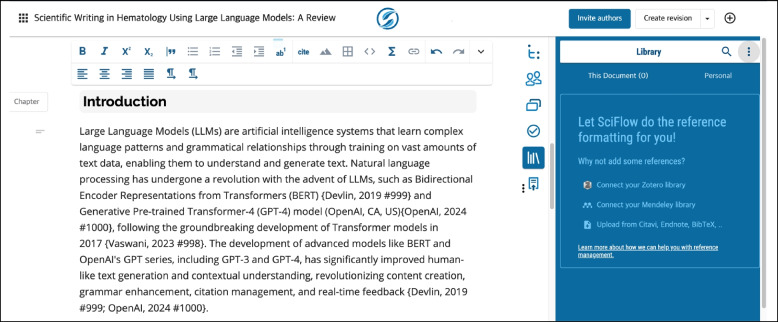
Usage of the SciFlow reference management interface. SciFlow web editor interface demonstrating manuscript editing tools and reference management panel. The right sidebar displays options for connecting reference libraries (Zotero, Mendeley) and automated citation formatting features

The market for AI tools is highly competitive, with new tools employing LLMs introduced weekly, many aiming to enhance the efficiency of scientific manuscript writing [40]. In general, these tools assist in grammar correction and enhance the readability of written texts. In addition to language enhancement, AI-powered tools have been developed to streamline the literature review process. For instance, Scispace (Typeset Inc., California, USA, https://scispace.com) facilitates paper-specific discussions, whereas Consensus (Consensus Science Inc., San Francisco, USA, https://consensus.app), Elicit (Elicit Research PBC, Oakland, CA, USA, https://elicit.com/), and Rayyan (Qatar Computing Research Institute, Doha, Qatar, https://rayyan.ai) provide customized search capabilities, paper summarizations, and systematic review support. Even though these tools accelerate research synthesis and writing discussions, researchers must maintain a critical oversight of AI-generated content. Connected Papers (Connected Papers Ltd., Tel Aviv, Israel, https://www.connectedpapers.com/) is a visualization tool that generates interactive maps of related academic papers, aiding researchers to visually explore literature relationships and research trends. These platforms employ advanced algorithms to extract and synthesize information from large volumes of scientific literature, thereby significantly reducing the time required for comprehensive literature reviews while maintaining high standards of accuracy and completeness. Tools such as SciFlow (SciFlow GmbH, Berlin, Germany, https://sciflow.net) streamline the writing process by providing real-time suggestions for structuring sections, managing references, and tracking revisions (Fig. 5).

### Quality assurance in LLM-assisted scientific writing

The integration of LLMs in Scientific writing requires robust quality assurance protocols to ensure scientific accuracy and reliability [41]. Although these advanced AI tools offer significant advantages in manuscript preparation and research synthesis, their outputs require careful systematic verification [34]. This verification becomes particularly crucial given the inherent limitations of LLMs, specifically their knowledge cut-off dates, which may prevent them from being aware of recent scientific developments. The dynamic nature of hematological research, characterized by rapid advances in treatment protocols and diagnostic methodologies, makes this additional verification process particularly important.

The verification of primary source citations has emerged as a critical component of quality assurance. LLMs may occasionally generate inaccurate citations or nonexistent reference sources, necessitating a thorough verification of all citations in the primary literature. Moreover, AI-generated literature reviews can result in the misconception or exclusion of key aspects or the exaggeration of insignificant details, potentially distorting the overall message and leading to flawed conclusions. Researchers should establish systematic workflows that incorporate multiple levels of verification, including the peer review of LLM-generated content, expert consultation for specialized topics, and comprehensive fact-checking against reliable scientific databases. This multi-layered approach has potential inaccuracies, outdated information, and methodological inconsistencies arising from the LLM assistance. The ultimate responsibility for scientific accuracy and integrity remains with human researchers despite the sophisticated capabilities of LLMs. Researchers must approach LLM assistance as a tool that augments human expertise and judgment instead of replacing scientific writing practices.

### Journal guidelines for AI usage in scientific writing

Ethical guidelines from academic journals and publishers provide specific instructions on the use of GAI in scholarly studies [42]. Some guidelines restrict GAI usage to the writing process, whereas others extend it to image generation and data analysis [43]. Publishers and journals emphasize that authors hold full responsibility for GAI-generated content, although the extent of this emphasis may vary. Ethical considerations highlight risks, such as inaccuracies, biases, and potential plagiarism, associated with GAI outputs [44]. Given the rapid evolution of GAI technology, guidelines must be continuously reviewed and updated, with increasing attention to their role in image generation and review processes. The academic community plays a crucial role in developing GAI usage policies, ensuring ethical compliance, and alerting and guiding authors on responsible use [45]. Ongoing review and adaptation of guidelines are essential to stay aligned with technological advancements and maintain the integrity of academic publishing [45].

A fundamental principle established across these journals is that AI systems cannot be recognized as manuscript authors as they cannot assume responsibility for scientific claims or research integrity. Additionally, researchers must explicitly disclose any use of AI tools in manuscript preparation, ensuring transparency in the research communication process; a position paper from the Committee on Publication Ethics Council provides details on disclosure and liability [46]. The scope of permitted AI utilization is usually defined by journal policies, with varying restrictions among publications [42].

## Discussion

The integration of AI into academic writing enhances productivity and expands the scope of content creation, streamlining the process for researchers [40]. LLMs can stimulate creative ideas within a specific subject and offer complementary information from the existing literature [38]. AI has become an integral part of modern daily life, and researchers should understand the fundamentals of this technology to harness its benefits.

LLMs can be effectively utilized in various academic tasks such as idea brainstorming, literature reviews, writing and proofreading academic submissions, programming, and coding support, structuring research outputs, data analysis, and generating visual content. These applications help to streamline the research process by enhancing the efficiency of exploring research topics, summarizing and analyzing data, improving writing quality, resolving coding errors, and organizing project outlines. Through carefully crafted prompting strategies and systematic frameworks, LLMs can significantly improve research efficiency across various tasks ranging from literature reviews to data analyses. The frameworks demonstrated for both manuscript development and data analysis highlight how LLMs can serve as powerful assistants while maintaining scientific rigor and domain expertise.

However, several limitations and challenges must be considered when implementing LLMs in research workflows. The application of LLMs presents significant challenges regarding accuracy and reliability as AI-generated content may contain errors or misinterpretations, particularly in the form of hallucinations, in which models produce plausible but incorrect information [47]. These accuracy concerns are particularly critical in medical fields, where inaccuracies could have serious implications for patient care [48, 49] and are particularly pronounced in complex tasks, such as statistical analyses and code generation. Additionally, privacy concerns and journal policies regarding AI usage present significant considerations that must be carefully addressed [50].

Additionally, while LLMs can assist with various aspects of research, they should complement, not replace, human expertise and critical thinking. Researchers must maintain careful oversight of LLM-generated content and ensure the appropriate validation of results, especially in clinical research contexts. These capabilities can relieve researchers of their cognitive burden, allowing for the allocation of more resources to the creative and analytical aspects of their work. Despite its potential, the adoption of AI in scientific writing has raised important ethical and practical concerns. Concerns regarding plagiarism, data privacy, and excessive dependence on AI-generated content highlight the need for clear guidelines and consensus on best practices [46].

Furthermore, AI systems are unable to remove the fundamental biases included within the training data, potentially leading to skewed results, and possibly exacerbating the imbalance throughout academic discourse. These challenges are compounded by ethical considerations regarding authorship attribution [43], plagiarism, and protection of data privacy. Moreover, challenges are compounded by the current lack of comprehensive regulatory frameworks governing AI usage in academic writing, although 87% of the 100 largest journals guide the use of GAI [42]. This necessitates the development of robust educational programs for responsible AI tool usage and the establishment of standardized ethical guidelines to ensure effective, safe, and sound utilization of AI technology for scientific writing.

## Conclusion

LLMs represent a transformative technology in hematological research and scientific writing that offers unprecedented support throughout the entire research lifecycle. Through effective prompt engineering and integration of RAG frameworks, these tools significantly enhance research efficiency in the literature review, data analysis, and manuscript preparation. The implementation of structured, prompt engineering strategies, combined with careful attention to scientific accuracy and methodological rigor, enables researchers to maximize the benefits of these advanced AI tools while maintaining high standards of research quality. However, the successful integration of LLMs in hematological research requires careful consideration of the ethical implications and adherence to established guidelines. Researchers must maintain strict data protection protocols, ensure transparent documentation of AI assistance, and comply with journal-specific requirements for AI usage. The response of the scientific community to LLM integration has been reflected in major journals.

The continued development of LLMs and their integration into research workflows presents both opportunities and challenges for the hematology community. Although these tools offer significant potential for enhancing research productivity and analytical capabilities, their successful implementation depends on the researchers' ability to effectively utilize them while maintaining scientific rigor and ethical standards. The future of hematological research will likely be shaped by the thoughtful integration of these technologies, guided by robust quality assurance frameworks and the evolution of best practices in scientific writing and research methodologies.

## Supplementary Information

Supplementary Material 1.

## Data Availability

No datasets were generated or analysed during the current study.
