# Supplementary material for: Transforming hematological research documentation with large language models: an approach to scientific writing and data analysis
Source: Blood Res. 2025 Mar 6;60(1):15. doi: 10.1007/s44313-025-00062-w (PMC11885755; doi:10.1007/s44313-025-00062-w)
Supplement: Supplementary file 1 — Supplementary Material 1. [file 44313_2025_62_MOESM1_ESM.zip › Supplementary Table S1-hsh.docx]

Supplementary Table S1.
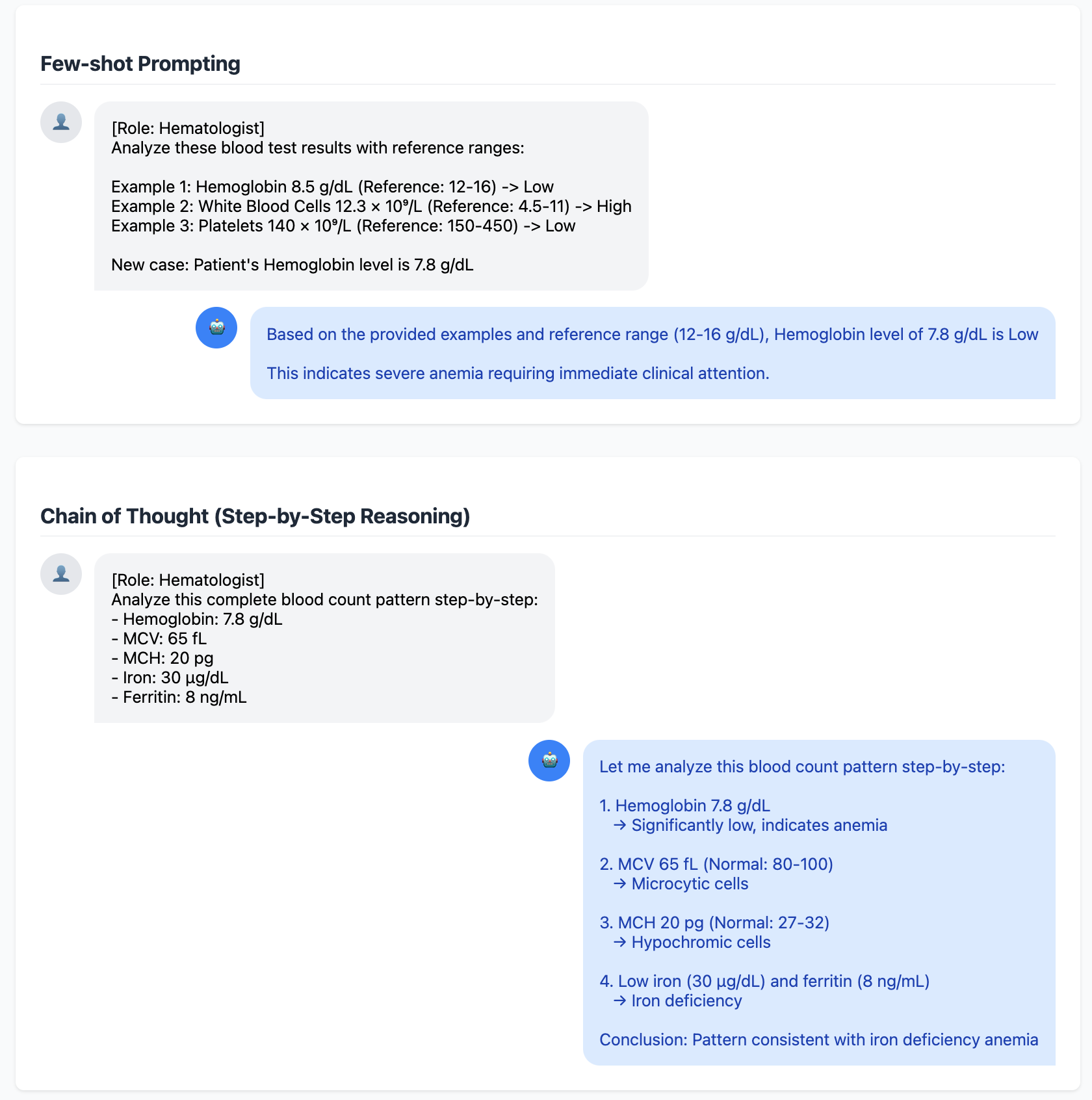


<!DOCTYPE html>

<html lang="en">

<head>

<meta charset="UTF-8">

<meta name="viewport" content="width=device-width, initial-scale=1.0">

<title>Enhanced Prompting Strategy Examples</title>

<style>

body {

margin: 0;

padding: 1.5rem;

background-color: #f9fafb;

font-family: -apple-system, BlinkMacSystemFont, 'Segoe UI', Roboto, sans-serif;

}

.container {

max-width: 64rem;

margin: 0 auto;

padding: 1.5rem;

}

.grid {

display: grid;

gap: 2rem;

}

.card {

background: white;

border-radius: 0.5rem;

padding: 1.5rem;

box-shadow: 0 1px 3px 0 rgba(0, 0, 0, 0.1);

}

.card-title {

font-size: 1.25rem;

font-weight: bold;

color: #1f2937;

padding-bottom: 0.5rem;

margin-bottom: 1rem;

border-bottom: 1px solid #e5e7eb;

}

.conversation {

display: flex;

flex-direction: column;

gap: 1rem;

}

.message {

display: flex;

align-items: flex-start;

gap: 0.75rem;

max-width: 100%;

}

.message.assistant {

flex-direction: row-reverse;

}

.icon {

width: 2.5rem;

height: 2.5rem;

border-radius: 9999px;

display: flex;

align-items: center;

justify-content: center;

flex-shrink: 0;

}

.user-icon {

background-color: #e5e7eb;

color: #4b5563;

}

.assistant-icon {

background-color: #3b82f6;

color: white;

}

.bubble {

max-width: 80%;

padding: 1rem;

white-space: pre-wrap;

border-radius: 1rem;

}

.user .bubble {

background-color: #f3f4f6;

border-bottom-left-radius: 0;

}

.assistant .bubble {

background-color: #dbeafe;

border-bottom-right-radius: 0;

color: #1e40af;

}

</style>

</head>

<body>

<div class="container">

<div class="grid" id="examples-container"></div>

</div>

<script>

const examples = [

{

title: "Few-shot Prompting",

conversation: [

{

role: "user",

text: "[Role: Hematologist]\nAnalyze these blood test results with reference ranges:\n\nExample 1: Hemoglobin 8.5 g/dL (Reference: 12-16) -> Low\nExample 2: White Blood Cells 12.3 × 10⁹/L (Reference: 4.5-11) -> High\nExample 3: Platelets 140 × 10⁹/L (Reference: 150-450) -> Low\n\nNew case: Patient's Hemoglobin level is 7.8 g/dL"

},

{

role: "assistant",

text: "Based on the provided examples and reference range (12-16 g/dL), Hemoglobin level of 7.8 g/dL is Low\n\nThis indicates severe anemia requiring immediate clinical attention."

}

]

},

{

title: "Chain of Thought (Step-by-Step Reasoning)",

conversation: [

{

role: "user",

text: "[Role: Hematologist]\nAnalyze this complete blood count pattern step-by-step:\n- Hemoglobin: 7.8 g/dL\n- MCV: 65 fL\n- MCH: 20 pg\n- Iron: 30 µg/dL\n- Ferritin: 8 ng/mL"

},

{

role: "assistant",

text: "Let me analyze this blood count pattern step-by-step:\n\n1. Hemoglobin 7.8 g/dL\n → Significantly low, indicates anemia\n\n2. MCV 65 fL (Normal: 80-100)\n → Microcytic cells\n\n3. MCH 20 pg (Normal: 27-32)\n → Hypochromic cells\n\n4. Low iron (30 µg/dL) and ferritin (8 ng/mL)\n → Iron deficiency\n\nConclusion: Pattern consistent with iron deficiency anemia"

}

]

}

];

function createMessageElement(message) {

const messageDiv = document.createElement('div');

messageDiv.className = `message ${message.role}`;

if (message.role === 'user') {

messageDiv.innerHTML = `

<div class="icon user-icon">👤</div>

<div class="bubble">${message.text}</div>

`;

} else {

messageDiv.innerHTML = `

<div class="bubble">${message.text}</div>

<div class="icon assistant-icon">🤖</div>

`;

}

return messageDiv;

}

function createExampleCard(example) {

const card = document.createElement('div');

card.className = 'card';

const title = document.createElement('h3');

title.className = 'card-title';

title.textContent = example.title;

card.appendChild(title);

const conversation = document.createElement('div');

conversation.className = 'conversation';

example.conversation.forEach(message => {

conversation.appendChild(createMessageElement(message));

});

card.appendChild(conversation);

return card;

}

const container = document.getElementById('examples-container');

examples.forEach(example => {

container.appendChild(createExampleCard(example));

});

</script>

</body>

</html>
