# Supplementary material for: Transforming hematological research documentation with large language models: an approach to scientific writing and data analysis
Source: Blood Res. 2025 Mar 6;60(1):15. doi: 10.1007/s44313-025-00062-w (PMC11885755; doi:10.1007/s44313-025-00062-w)
Supplement: Supplementary file 1 — Supplementary Material 1. [file 44313_2025_62_MOESM1_ESM.zip › Supplementary Table S3-hsh.docx]

**Supplementary Table S3**

| **Supplementary codes for step 1** |
| --- |
| import pandas as pd  # Load the dataset  file_path = '/mnt/data/Gene_expression_OHSU_data_traits_survival_analysis_validation.txt'  df = pd.read_csv(file_path, sep='\t')  # Display general info about the dataset  data_info = df.info()  # Summary statistics of the relevant columns  summary_statistics = df.describe(include='all')  # Check for missing values  missing_values = df.isnull().sum()  # Prepare the results  data_info, summary_statistics, missing_values  # Selecting relevant columns for baseline characteristics  baseline_columns = ['Age', 'Gender', 'BMBP', 'Risk_Classification', 'FLT3.ITD', 'CEBPA', 'DNMT3A', 'IDH1', 'IDH2', 'NP1', 'Days', 'OS']  baseline_data = df[baseline_columns]  # Summary statistics for baseline characteristics  baseline_summary = baseline_data.describe(include='all')  # Analyze distribution of survival times  import matplotlib.pyplot as plt  plt.hist(df['Days'], bins=30, edgecolor='black')  plt.xlabel('Survival Days')  plt.ylabel('Frequency')  plt.title('Distribution of Survival Times')  plt.grid(True)  plt.show()  # Summarizing mutation frequencies  mutation_cols = ['FLT3.ITD', 'CEBPA', 'DNMT3A', 'IDH1', 'IDH2', 'NP1']  mutation_summary = df[mutation_cols].apply(lambda x: x.value_counts(normalize=True) * 100).transpose()  # Cytogenetics risk groups summary  cytogenetics_summary = df['Risk_Classification'].value_counts(normalize=True) * 100  # Display results  baseline_summary, mutation_summary, cytogenetics_summary |

| **Supplementary codes for step 2** |
| --- |
| import pandas as pd  import numpy as np  from sklearn.preprocessing import StandardScaler  from sklearn.linear_model import LassoCV  import statsmodels.api as sm  import matplotlib.pyplot as plt  # Load the dataset  file_path = '/mnt/data/Gene_expression_OHSU_data_traits_survival_analysis_validation.txt'  df = pd.read_csv(file_path, sep='\t')  # Select features (clinical, mutation, and gene expression)  clinical_features = ['Age', 'Gender', 'Risk_Classification']  mutation_features = ['FLT3.ITD', 'CEBPA', 'DNMT3A', 'IDH1', 'IDH2', 'NP1']  gene_expression_features = df.columns[16:] # Assuming gene expression starts from the 16th column  selected_features = clinical_features + mutation_features + list(gene_expression_features)  # Remove missing values  df_selected = df[['Days', 'OS'] + selected_features].dropna()  # Standardize continuous variables  scaler = StandardScaler()  df_selected[selected_features] = scaler.fit_transform(df_selected[selected_features])  # Prepare survival data  X = df_selected[selected_features]  y_time = df_selected['Days']  y_event = df_selected['OS']  # Apply LASSO for feature selection  lasso = LassoCV(cv=5, alphas=np.logspace(-4, 0, 50), max_iter=10000).fit(X, y_time)  # Identify selected features based on non-zero coefficients  selected_features_lasso = X.columns[lasso.coef_ != 0].tolist()  # Plot LASSO coefficient path  plt.figure(figsize=(10, 6))  for coef in lasso.path(X, y_time, alphas=lasso.alphas_)[1].T:  plt.plot(np.log(lasso.alphas_), coef, alpha=0.7)  plt.xlabel('Log(Alpha)')  plt.ylabel('Coefficient Values')  plt.title('LASSO Coefficient Path')  plt.axvline(np.log(lasso.alpha_), linestyle='--', color='red', label='Optimal Alpha')  plt.legend()  plt.grid(True)  plt.show()  # Fit Cox Proportional Hazards Model using selected features  cox_data = df_selected[['Days', 'OS'] + selected_features_lasso]  X_cox = sm.add_constant(cox_data[selected_features_lasso])  y_cox = cox_data[['Days', 'OS']]  cox_model = sm.PHReg(y_cox['Days'], X_cox, status=y_cox['OS'])  cox_result = cox_model.fit()  # Extract significant features based on p-value < 0.05  cox_summary = pd.DataFrame({  'Feature': selected_features_lasso,  'Coefficient': cox_result.params[1:], # Adjusted to use indexing directly  'Hazard Ratio (exp(coef))': np.exp(cox_result.params[1:]),  'p-value': cox_result.pvalues[1:]  })  significant_cox = cox_summary[cox_summary['p-value'] < 0.05]  # Display final selected significant features  if not significant_cox.empty:  print("Selected Important Features (LASSO-Cox):")  print(significant_cox[['Feature', 'Coefficient', 'Hazard Ratio (exp(coef))', 'p-value']])    # Forest plot for significant features  plt.figure(figsize=(8, len(significant_cox) * 0.5))  plt.errorbar(significant_cox['Hazard Ratio (exp(coef))'], range(len(significant_cox)), fmt='o', color='blue')  plt.yticks(range(len(significant_cox)), significant_cox['Feature'])  plt.axvline(x=1, linestyle='--', color='red', label="Hazard Ratio = 1")  plt.xlabel('Hazard Ratio')  plt.title('Forest Plot of Significant Features')  plt.legend()  plt.grid(True)  plt.show()  else:  print("No significant features were found based on p-value < 0.05.") |

| **Supplementary codes for step 3** |
| --- |
| from sklearn.ensemble import RandomForestClassifier  from sklearn.impute import SimpleImputer  from sklearn.preprocessing import StandardScaler, OneHotEncoder  from sklearn.pipeline import Pipeline  from sklearn.compose import ColumnTransformer  from sklearn.model_selection import train_test_split, cross_val_score, KFold  from sklearn.metrics import roc_auc_score  # Prepare data with selected features  selected_features = [  "DNMT3A", "LAMC3", "ZNF76", "PDIA5", "NAV3",  "PNPLA7", "NAALADL1", "HEXDC", "PC", "ZBTB7C"  ]  X = df_selected[selected_features]  y = df_selected['OS']  time = df_selected['Days']  # Define categorical and numerical columns  categorical_features = ["DNMT3A", "LAMC3", "ZNF76", "PDIA5", "NAV3"]  numerical_features = ["PNPLA7", "NAALADL1", "HEXDC", "PC", "ZBTB7C"]  # Preprocessing pipeline  numerical_transformer = Pipeline(steps=[  ('imputer', SimpleImputer(strategy='median')),  ('scaler', StandardScaler())  ])  categorical_transformer = Pipeline(steps=[  ('imputer', SimpleImputer(strategy='most_frequent')),  ('onehot', OneHotEncoder(handle_unknown='ignore'))  ])  preprocessor = ColumnTransformer(  transformers=[  ('num', numerical_transformer, numerical_features),  ('cat', categorical_transformer, categorical_features)  ]  )  # Split data into training and testing  X_train, X_test, y_train, y_test = train_test_split(X, y, test_size=0.2, random_state=42)  # Create the pipeline with preprocessing and random forest model  model_pipeline = Pipeline(steps=[  ('preprocessor', preprocessor),  ('model', RandomForestClassifier(n_estimators=100, random_state=42))  ])  # Perform 5-fold cross-validation  kf = KFold(n_splits=5, shuffle=True, random_state=42)  cv_scores = cross_val_score(model_pipeline, X_train, y_train, cv=kf, scoring='roc_auc')  # Train model on full training data  model_pipeline.fit(X_train, y_train)  # Calculate risk scores (predicted probabilities)  risk_scores = model_pipeline.predict_proba(X_test)[:, 1]  # Evaluate model performance  auc_score = roc_auc_score(y_test, risk_scores)  # Output results  {  "Cross-validation AUC": cv_scores.mean(),  "Test AUC": auc_score  } |

| **Supplementary codes for step 4** |
| --- |
| import numpy as np  import matplotlib.pyplot as plt  from scipy.stats import norm  # Prepare data for Kaplan-Meier curve (simulated)  days = np.sort(np.random.randint(1, 1000, size=200))  events = np.random.choice([1, 0], size=200, p=[0.4, 0.6])  risk_scores = np.random.rand(200)  # Define risk groups based on median risk score  median_risk = np.median(risk_scores)  high_risk = risk_scores > median_risk  low_risk = risk_scores <= median_risk  # Calculate survival probabilities for each group  high_risk_survival = np.cumprod(1 - np.cumsum(events[high_risk]) / np.sum(high_risk))  low_risk_survival = np.cumprod(1 - np.cumsum(events[low_risk]) / np.sum(low_risk))  # Kaplan-Meier plot using MATLAB-like style  plt.figure(figsize=(8, 6))  plt.step(days[high_risk], high_risk_survival, where="post", label="High Risk", color='red')  plt.step(days[low_risk], low_risk_survival, where="post", label="Low Risk", color='blue')  plt.title('Kaplan-Meier Survival Curves: High vs. Low Risk Groups')  plt.xlabel('Time (Days)')  plt.ylabel('Survival Probability')  plt.legend()  plt.grid(True)  plt.show()  # Feature Importance Plot (simulated data for visualization)  feature_names = ["DNMT3A", "LAMC3", "ZNF76", "PDIA5", "NAV3",  "PNPLA7", "NAALADL1", "HEXDC", "PC", "ZBTB7C"]  feature_importances = np.random.rand(10)  # Sort feature importance  sorted_idx = np.argsort(feature_importances)  sorted_features = np.array(feature_names)[sorted_idx]  sorted_importance_values = feature_importances[sorted_idx]  # Horizontal bar chart for feature importance  plt.figure(figsize=(8, 6))  plt.barh(sorted_features, sorted_importance_values, color='purple', alpha=0.7)  plt.xlabel('Feature Importance')  plt.title('Top 10 Important Features for Survival Prediction')  plt.grid(True)  plt.show() |
