# Supplementary material for: Transforming hematological research documentation with large language models: an approach to scientific writing and data analysis
Source: Blood Res. 2025 Mar 6;60(1):15. doi: 10.1007/s44313-025-00062-w (PMC11885755; doi:10.1007/s44313-025-00062-w)
Supplement: Supplementary file 1 — Supplementary Material 1. [file 44313_2025_62_MOESM1_ESM.zip › Supplementary Table S4-hsh.docx]

Supplementary Table S4– RNAseq data analysis

| User:  I have obtained bulk RNA-seq data and would like to perform a comprehensive analysis to identify a set of significantly differentially expressed genes (DEGs). Could you provide a detailed framework or step-by-step process covering the following key aspects? |
| --- |
| ChatGPT 4o:  Certainly! Here’s a comprehensive step-by-step framework to analyze bulk RNA-seq data for identifying differentially expressed genes (DEGs):  1. Data Preprocessing  1.1. Quality Control (QC)  Tools: FastQC, MultiQC  Steps:  Assess read quality (e.g., per base sequence quality, adapter contamination).  Check for GC content, sequence duplication levels, and overrepresented sequences.  Generate an aggregated report using MultiQC.  1.2. Read Trimming  Tools: Trim Galore, Cutadapt  Steps:  Remove low-quality bases.  Trim adapters.  Discard reads below a certain length threshold.  2. Alignment and Quantification  2.1. Read Alignment (or Pseudo-alignment)  Alignment-based tools: STAR, HISAT2  Alignment-free tools: Salmon, Kallisto  Steps:  Align reads to the reference genome (e.g., GRCh38 for humans).  Generate alignment metrics (e.g., mapping rate, read distribution across exons/introns).  Convert SAM to BAM files and sort them using SAMtools.  2.2. Expression Quantification  Tools: FeatureCounts (for alignment-based), Salmon/Kallisto (for pseudo-alignment)  Steps:  Count reads mapped to genes or transcripts.  Generate count matrices for downstream analysis.  3. Exploratory Data Analysis (EDA)  3.1. Quality Control at Expression Level  Tools: DESeq2, edgeR, limma (in R)  Steps:  Assess library size and expression distributions.  Visualize raw data using boxplots and density plots.  Identify and remove lowly expressed genes.  3.2. Sample Clustering and Outlier Detection  Tools: PCA, t-SNE, UMAP  Steps:  Perform Principal Component Analysis (PCA) to detect batch effects.  Identify outliers or batch effects using hierarchical clustering.  Consider normalization methods to reduce variability.  4. Differential Expression Analysis  4.1. Normalization  Tools: DESeq2 (median ratio normalization), edgeR (TMM normalization)  Steps:  Normalize raw counts to account for sequencing depth and gene length.  Apply variance stabilizing transformation (VST) or regularized log transformation (rlog).  4.2. DEG Identification  Tools: DESeq2, edgeR, limma-voom  Steps:  Design an experimental model (e.g., condition + batch effects).  Perform statistical testing (Wald test or likelihood ratio test).  Adjust for multiple testing (Benjamini-Hochberg FDR correction).  Criteria for DEGs:  Fold Change (e.g., \|log2FC\| > 1)  Adjusted p-value (e.g., < 0.05)  4.3. Visualization  Tools: ggplot2 (R), ComplexHeatmap, EnhancedVolcano  Steps:  Volcano plot for DEGs visualization.  Heatmap of top DEGs with hierarchical clustering.  MA plot to assess global expression changes.  5. Functional Enrichment Analysis  5.1. Pathway and Gene Ontology (GO) Analysis  Tools: clusterProfiler, gProfiler, DAVID, Enrichr  Steps:  Identify enriched pathways (KEGG, Reactome).  Perform GO enrichment (Biological Process, Molecular Function, Cellular Component).  Visualize enriched terms using bar plots or dot plots.  5.2. Gene Set Enrichment Analysis (GSEA)  Tools: GSEA (Broad Institute), fgsea (R package)  Steps:  Rank genes based on log2FC and significance.  Identify enriched pathways using curated gene sets (MSigDB).  Plot enrichment curves for significant pathways.  6. Additional Analyses  6.1. Gene Co-expression Network Analysis  Tools: WGCNA (R package)  Steps:  Construct co-expression modules.  Identify module-trait relationships.  Annotate key hub genes in significant modules.  6.2. Survival and Clinical Correlation Analysis (if clinical data available)  Tools: Survival package (R), Cox regression analysis  Steps:  Integrate gene expression with survival data.  Perform Kaplan-Meier survival analysis.  7. Integration with Other Omics Data (Optional)  Tools: MultiOmics packages (MOFA, iCluster)  Steps:  Integrate RNA-seq with proteomics, methylation, or single-cell data.  Explore cross-omics relationships.  8. Reporting and Documentation  8.1. Results Interpretation  Summarize key findings with biological context.  Highlight the most promising genes/pathways.  8.2. Reproducibility  Tools: Snakemake, Nextflow for pipeline automation.  Save scripts, metadata, and intermediate results.  Use version control (GitHub, Zenodo) for tracking changes.  9. Validation (Optional)  Experimental Validation: qPCR, Western blot, IHC.  In-silico Validation: External datasets (e.g., TCGA, GEO).  Recommended Workflow Tools  RNA-seq pipeline tools: nf-core RNA-seq, bcbio-nextgen.  Interactive tools: RStudio, Jupyter Notebook for documentation.  Would you like assistance in implementing a specific part of the analysis or recommendations on tools for automation? |
